# Organizational Effects of Estrogens and Androgens on Estrogen and Androgen Receptor Expression in Pituitary and Adrenal Glands in Adult Male and Female Rats

**DOI:** 10.3389/fnana.2022.902218

**Published:** 2022-06-23

**Authors:** Natalia Lagunas, José Manuel Fernández-García, Noemí Blanco, Antonio Ballesta, Beatriz Carrillo, Maria-Angeles Arevalo, Paloma Collado, Helena Pinos, Daniela Grassi

**Affiliations:** ^1^Department of Legal Medicine, Psychiatry and Pathology, School of Medicine, Complutense University of Madrid, Madrid, Spain; ^2^Department of Psychobiology, National University of Distance Education, Madrid, Spain; ^3^Department of Psychology, Universidad Villanueva, Madrid, Spain; ^4^Department of Psychology, Faculty of Biomedical Science and Health, European University of Madrid, Madrid, Spain; ^5^University Institute of Research-UNED-Institute of Health Carlos III (IMIENS), Madrid, Spain; ^6^Neuroactive Steroids Lab, Cajal Institute, CSIC, Madrid, Spain; ^7^Centro de Investigación Biomédica en Red de Fragilidad y Envejecimiento Saludable (CIBERFES), Instituto de Salud Carlos III, Madrid, Spain; ^8^Department of Anatomy, Histology and Neuroscience, Autonomous University of Madrid, Madrid, Spain

**Keywords:** estrogen receptor, androgen receptor, GPER, pituitary, adrenal gland, organizational effect, estradiol, testosterone

## Abstract

Sex steroid hormones, such as androgens and estrogens, are known to exert organizational action at perinatal periods and activational effects during adulthood on the brain and peripheral tissues. These organizational effects are essential for the establishment of biological axes responsible for regulating behaviors, such as reproduction, stress, and emotional responses. Estradiol (E2), testosterone, and their metabolites exert their biological action through genomic and non-genomic mechanisms, bounding to canonical receptors, such as estrogen receptor (ER)α, ERβ, and androgen receptor (AR) or membrane receptors, such as the G protein-coupled estrogen receptor (GPER), respectively. Expression of ERs and AR was found to be different between males and females both in the brain and peripheral tissues, suggesting a sex-dependent regulation of their expression and function. Therefore, studying the ERs and AR distribution and expression levels is key to understand the central and peripheral role of sex steroids in the establishment of sex-specific behaviors in males and females. We investigated the organizational effects of estrogens and androgens in the pituitary and adrenal glands of adult male and female rats. For this, selective blockade of AR with flutamide or 5α-reductase with finasteride or aromatase with letrozole during the first 5 days of life has been performed in male and female pups and then quantification of ERs and AR expression in both glands has been carried out in adulthood. Data show that inhibition of dihydrotestosterone (DHT) and E2 production during the first five postnatal days mainly decreases the ER expression in male to female values and AR expression in female to male levels in the pituitary gland and increases AR expression in female to male levels in the adrenal gland. In contrast, blocking the action of androgens differentially modulates the ERs in males and females and decreases AR in both males and females in both glands. Altogether, the results suggest that neonatal modifications of the androgen and estrogen pathways can potentially lead to permanent modifications of the neuroendocrine functions of the pituitary and adrenal glands in the adulthood of both sexes.

## Introduction

Steroid hormones are a group of molecules that derive from the cholesterol by a biochemical process known as steroidogenesis, occurring in several tissues, such as gonads, brain, and adrenal glands, among others. In addition, they impact on physiology and behavior by two effects, namely, an organizational effect on critical developmental moments, such as prenatal and postnatal first days after birth, to promote sex brain differences, and an activational effect occurring in adulthood, contributing to behaviors and functions with hormonal implications, such as sexual behavior, stress response, and emotions (Arnold and Breedlove, 1985; Cooke et al., 1998). Along the steroidogenesis, testosterone is converted into estradiol (E2) by aromatase or metabolized to dihydrotestosterone (DHT) by 5α-reductase. Testosterone can then act throughout the body either *via* direct action on androgen receptors (ARs) or through its metabolites, DHT, and E2, which are able to interact with ARs or estrogen receptors (ERs), respectively (Zuloaga et al., 2020). Estrogen and testosterone act by genomic (i.e., into the nucleus binding ERα, ERβ, and AR) and non-genomic mechanisms (i.e., binding membrane receptors and activating second-messenger cascades) (Foradori et al., 2008) exerting organizational and activational effects in the brain and throughout the whole body. Studying the distribution of ERs and AR is key to understand the central and peripheral role of sex steroids. Several structures have been reported to express ERs and AR, such as cerebral cortex, amygdaloid complex, hypothalamus, hippocampus, pituitary, thyroid, adrenal glands, and gonads, among others (Pelletier, 2000; Xiao and Jordan, 2002; Llorente et al., 2020; Marraudino et al., 2021).

Organizational effects of sex steroid hormones during the perinatal life have major irreversible effects on adults defining, since very early in the postnatal development, sex-specific biological features reflect sex-specific behaviors and physiology. E2, testosterone, and their metabolites and signaling pathways are involved in cellular growth, neurotransmission, and neuroprotection (Hsu et al., 2001; Mukai et al., 2007; Morissette et al., 2008; Bustamante-Barrientos et al., 2021), impacting in neuroendocrine system organization, particularly, on the hypothalamus and the limbic system (Matsumoto, 1991). Indeed, the organizational effects of sex steroid hormones have been largely described in the brain. Perinatal gonadectomy has been reported to reduce in adult male animals the volume of the sexually dimorphic nucleus of the medial preoptic area and ventromedial nucleus (Matsumoto and Arai, 1986), hypothalamic regions involved in the sexual behavior (Morishita et al., 2021), and neurohormonal food intake and glucose regulation, thermoregulation, and social and sexual behaviors (Khodai and Luckman, 2021), respectively. In addition, perinatal gonadectomy in male rats impairs the volume and number of neurons of several structures of the vomeronasal system (Guillamon and Segovia, 1997). Furthermore, limbic structures such as the amygdaloid complex, particularly the bed nucleus of the stria terminalis (Maita et al., 2021) participating in the regulation of the stress response (Maita et al., 2021) and reported to have greater volume in males than in females and larger corticotropin-releasing factor (CRF)-producing neurons in females than in males, have been shown to be affected by gonadectomy since it abolished these sexual differences (Del Abril et al., 1987; Uchida et al., 2019). These organizational effects of sex steroid hormones could underlie the sex differences that appear in the function of the hypothalamus–pituitary–adrenal axis (HPA) (Heck and Handa, 2019), the hypothalamus–pituitary–thyroid axis (HPT) (Parra-Montes De Oca et al., 2021), and the social behavior network (SBN) (Dovey and Vasudevan, 2020) implicated in behavioral, cognitive, and emotional processes. Unfortunately, very less is known about the gonadal hormones' organizational effects on the peripheral components of these neuroendocrine systems, such as in the pituitary and adrenal glands.

The pituitary is a tiny but essential gland located in the hypophyseal fossa at the base of the skull, anatomically, and functionally organized in two portions, namely, the anterior and posterior lobes. The pituitary anterior lobe (adenohypophysis) has the ectodermal origin and conveys numerous hypothalamic projections from the arcuate, ventromedial, dorsomedial, medial preoptic, and paraventricular (parvocellular) nuclei, among others, whereas the pituitary posterior lobe (neurohypophysis) with the diencephalic origin receives the hypothalamic projections originated in the supraoptic and magnocellular portions of the paraventricular nucleus. The anterior lobe is part of the parvocellular neuroendocrine system that controls the release of numerous hormones [e.g., growth hormone (GH), prolactin (PRL), melanocyte-stimulating hormone, follicle-stimulating hormone (FSH), luteinizing hormone (LH), and adrenocorticotropic hormone, among others], whereas the posterior lobe is part of the magnocellular neuroendocrine system that regulates the production and secretion of oxytocin and vasopressin (Dorton, 2000). Adrenal glands are also functionally and anatomically divided into two portions, namely, the adrenal medulla, innerved by preganglionic neurons exerting sympathetic stimulation over chromaffin cells and provoking catecholamines release, and adrenal cortex that synthesizes corticosteroids and androgens (Wehrwein et al., 2016). Organizational roles of estrogens and androgens on the pituitary and adrenal glands ontogeny are not fully understood yet, but some evidence highlights their role in the modulation of pituitary and adrenal functions. Indeed, perinatal exposure to androgens as well as to estrogens “masculinizes” corticosterone response in adulthood in females (Patchev et al., 1995; Seale et al., 2005a) bringing corticosterone levels in adult female to adult male values. In addition, neonatal gonadectomy in males “feminizes” HPA response and increases mineralocorticoid receptor binding in the pituitary (Mccormick et al., 1998) since deprivation of testosterone and E2 during perinatal periods increase corticosterone levels to female levels. Moreover, when low doses of testosterone are administrated perinatally, the effects on development and reproduction were no different, but corticosterone level in adulthood during stress response was significantly decreased in males (Wilson et al., 2020). In light of these data, organizational action of estrogens and androgens on the establishment of the pituitary and adrenal neuroendocrine systems in the adult male and female animals can be postulated. Interestingly, estrogen and androgen organizational action on ERs and AR expression levels in these glands has not been explored yet. We hypothesized a possible differential expression of ERs and AR in the pituitary and adrenal glands in male and female rats under specific programming effect of androgens and estrogens during the first postnatal critical period that could be at the base of the sex-dependent biological differences of the pituitary and adrenal gland in males and females. For this purpose, we investigated, in the pituitary and adrenal glands of adult male and female rats, the effects on ERs and AR expression levels of (I) the inhibition of the androgen pathway by selectively blocking the AR and (II) fostering the estrogen pathway by selectively inhibiting aromatase and 5α-reductase during the first 5 postnatal days.

## Materials and Methods

All experiments were designed according to the guidelines presented in the “Guidelines for the Use of Animals in Neuroscience Research” by the Society for Neuroscience, the European Union legislation (Council Directives 86/609/EEC and 2010/63/UE), and the Spanish Government Directive (R.D. 1201/2005). Experimental procedures were approved by our Institutional Bioethical Committee (UNED, Madrid). Special care was taken to minimize animal suffering and to reduce the number of animals used to the minimum necessary.

### Animals and Experimental Treatments

Wistar albino male and female rats from our in-house colony were kept on a 12:12-h light–dark cycle and received food and water *ad libitum*. For mating, a male was placed in a cage with two females for 1 week. Pregnant females were housed individually in plastic maternity cages with wood shavings as the nesting material. Twelve pregnant females were used in order that at least 2–3 mothers give birth within the same 24 h. On the postnatal day 1 (P1), pups from these litters born within the same 24 h were weighed, sexed, and randomly distributed to the different experimental conditions, assigning five females and five males to each mother.

**Figure 1:**
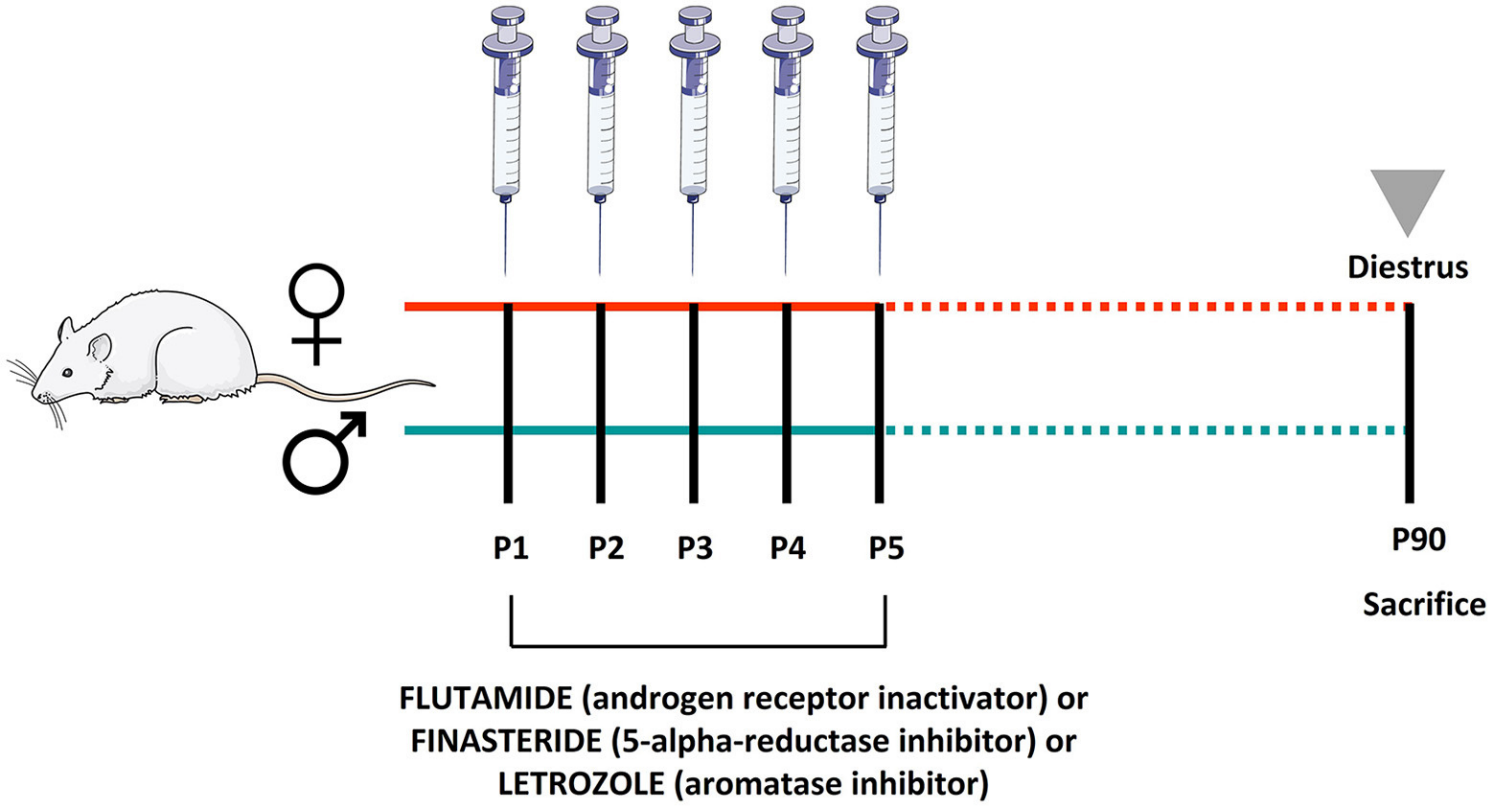
Schematic diagram of the experimental setup: male and female pups were treated during the first five postnatal days with flutamide, a selective androgen receptor inactivator, or finasteride, a selective 5α-reductase inhibitor, or letrozole, a selective aromatase inhibitor. Animals were sacrificed at the postnatal day 90.

From P1 to P5, pups received a daily s.c. injection of flutamide [(competitively blocks ARs, forming an inactive form that cannot translocate into the cell nucleus), 25 mg/kg, injected volume 0.01 ml/kg, F9397, Merk], or finasteride [(competitively inhibits the steroid type II 5-alpha-reductase interfering with the enzymatic conversion of testosterone to 5-DHT), 5 mg/kg, injected volume 0.01 ml/kg, NB-48-0403-100MG, Quimigen], or letrozole [(inhibits the aromatase), 1 mg/kg, injected volume 0.01 ml/kg, L6545, Merck]. Control animals were treated s.c. with vehicle (corn oil). The doses were selected based on the previous studies (Fanaei et al., 2013; Yamada et al., 2015; Cataldi et al., 2018; Cheng et al., 2019). On the postnatal day 90 (P90), rats were weighed and decapitated between 9:00 and 11:00 a.m. Females were then sacrificed during the diestrus phase. The diestrus phase was identified followed by the procedure previously described (Marcondes et al., 2002; Hubscher et al., 2005). A diagram with the experimental setup and molecular targets of the inhibitors is shown in Figure 1. Pituitary and adrenal glands were dissected, rapidly frozen in dry ice, and stored at −80°C.

### Quantitative Real-Time Polymerase Chain Reaction (PCR)

Total RNA was extracted from the isolated tissues with an illustra RNAspin mini-RNA isolation kit (GE Healthcare) followed by the manufacturer's instructions to measure ERα, ERβ, G protein-coupled estrogen receptor (GPER), and AR mRNA levels. 18S rRNA was selected as the control housekeeping gene. The first cDNA strand was synthesized using 2 μg of extracted RNA by reverse transcription in a final volume of 15 μl using the RevertAid H Minus first-strand cDNA synthesis kit (MBI Fermentas) following the manufacturer's instructions. After reverse transcription, the cDNA was diluted in a ratio of 1:10, and 5 μl were amplified by real-time PCR in 20 μl using SYBR Green master mix (Applied Biosystems) in an ABI Prism 7500 sequence detector (Applied Biosystems), with conventional Applied Biosystems cycling parameters (40 cycles of 95°C, 15 s, and 60°C, 1 min). All samples were amplified in duplicate. Primer sequences were designed using Primer Express (Applied Biosystems) and, after amplification, the size of the quantitative real-time PCR products was verified by electrophoresis on 2% (w/v) ethidium bromide-stained agarose gel, and the identity of PCR products was ascertained by sequencing. Bands were excised and cDNA was purified using the QIAquick PCR purification Kit (Quiagen Gmbg, Hilden, Germany). An amount of 100 ng ERα, ERβ, GPER, and AR cDNA samples were sequenced (Automatic Sequencing Center, CSIC, Madrid, Spain) with the same sense and antisense primers. The obtained sequence was aligned with the expected sequence obtained from the GenBank. In addition, dissociation curve analysis was also performed after each PCR reaction to ensure that a single product and no primer dimers were present. Primers for ERα, ERβ, GPER, AR, and 18S in rats were used as previously described by our group (Acaz-Fonseca et al., 2020), namely, ERα forward, 5′-GCCACTCGATCATTCGAGCA-3′ and reverse, 5′-CCTGCTGGTTCAAAAGCGTC-3′; ERβ forward, 5′-GCTGGGCCAAGAAAATCCCT-3′ and reverse, 5′- CCCCTCATCCCTGTCCAGAA−3′; GPER forward, 5′- GGACAAGCTCAGGCTGTATGTG−3′ and reverse, 5′- GCTCCGTGCTGTCTGGTATGA−3′; AR forward, 5′- AAAAGAGCTGCGGAAGGGAA−3′ and reverse, 5′- CATTTCCGGAGACGACACGA−3′ and 18S rRNA forward, 5′-CGCCGCTAGAGGTGAAATTCT-3′ and reverse, 5′-CATTCTTGGCAAATGCTTTCG-3′. The Ct was determined and normalized to the housekeeping gene 18 s rRNA, and the ΔΔCT method was used to determine relative expression levels. Statistics were performed using ΔΔCT threshold cycle values.

### Statistical Analysis

Statistical significance was assessed by two-way ANOVA to analyze the effect and the interaction between the variable “sex” and the variable “treatment” and by one-way ANOVA to analyze the receptors' relative amounts and treatment differences among other groups, followed by the Bonferroni's *post-hoc* test to compare group differences and *t*-test to analyze differences between control groups. Statistical analysis was performed using the SPSS version 25.0 software (SPSS Inc., Chicago, USA). The levels of significance were denoted as ^*^P < 0.05; ^**^P < 0.01; ^***^P < 0.001. A level of *P* < 0.05 was considered statistically significant. Levene's test was used to assess the equality of variances. Data in the figures are represented as mean ± SEM.

## Results

### Differential Estrogen and Androgen Receptors Expression in the Pituitary Gland

**Figure 2:**
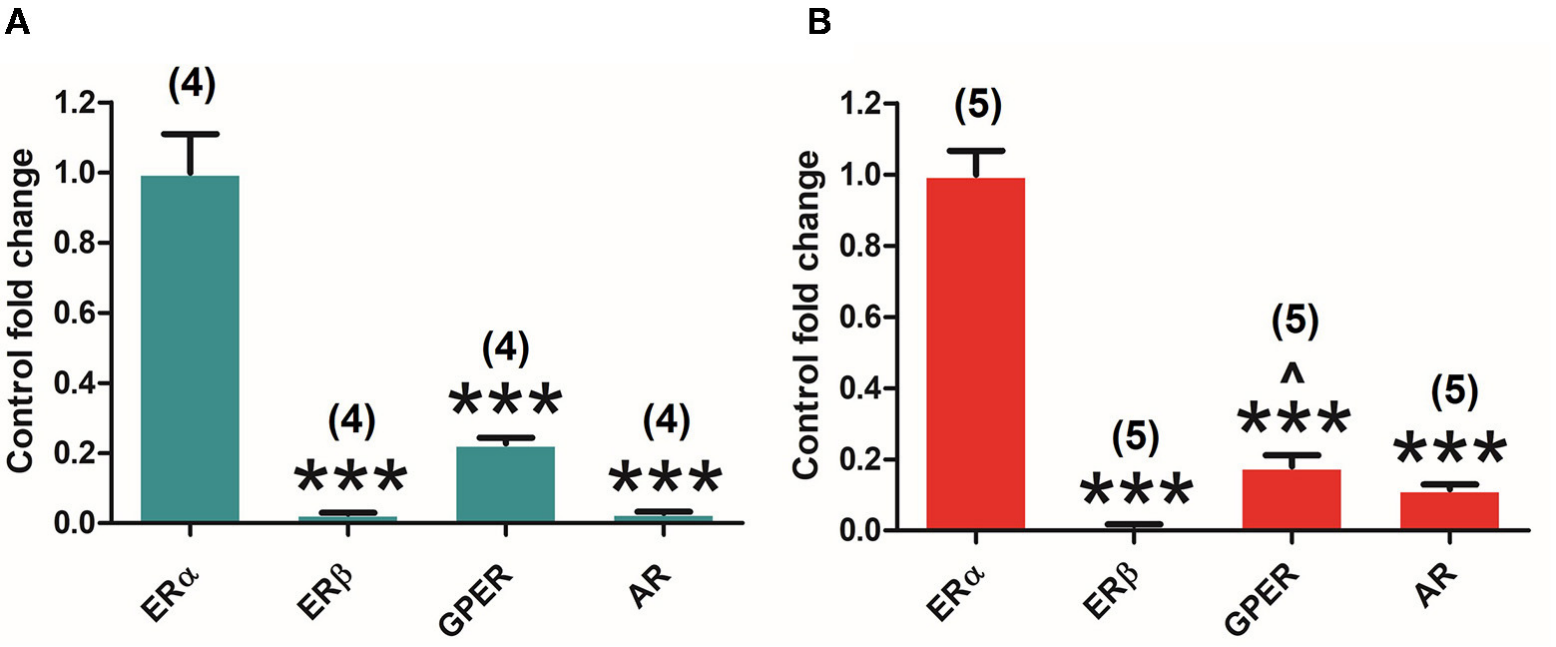
Differential estrogen receptor (ER) and androgen receptor (AR) expression in the pituitary gland of adult male and female rats. **(A)** ERα, ERβ, G protein-coupled estrogen receptor (GPER), and AR expression in male animals (*n* = 4). **(B)** ERα, ERβ, GPER, and AR expression in female animals (*n* = 5). Data are represented as mean ± SEM. ***Significant differences (***p < 0.001) vs. ERα values. ^∧^Significant differences (^∧^*p* < 0.05) vs. ERβ values.

Both canonical (ERα and ERβ) and GPER as well as AR are expressed in the pituitary gland of both adult male and female rats. Interestingly, the analysis of the different receptors' expression levels in the pituitary highlights the differences in their relative amount in both males and females. One-way ANOVA of the receptors' relative amounts revealed significant differences in males [*F*_(3, 12)_ = 69.309; *P* = 0.000], and subsequent Bonferroni's *post-hoc* test showed the ERα in comparison with ERβ (*P* = 0.000), GPER (*P* = 0.000), and AR (*P* = 0.000) (Figure 2A). Similar results were found in females. Indeed, ANOVA revealed significant differences [*F*_(3, 16)_ = 149.864; *P* = 0.000] and subsequent Bonferroni's *post-hoc* test revealed a higher amount of ERα compared to ERβ (*P* = 0.000), GPER (*P* = 0.000), and AR (*P* = 0.000), and a higher amount of GPER in comparison with ERβ (*P* = 0.037) (Figure 2B). In both male and female animals, ERα seems to be the highest and ERβ the lowest expressed, showing GPER and AR intermediate levels of expression.

### Sex Dimorphism in Estrogen and Androgen Receptor Expression in the Pituitary Gland

**Figure 3:**
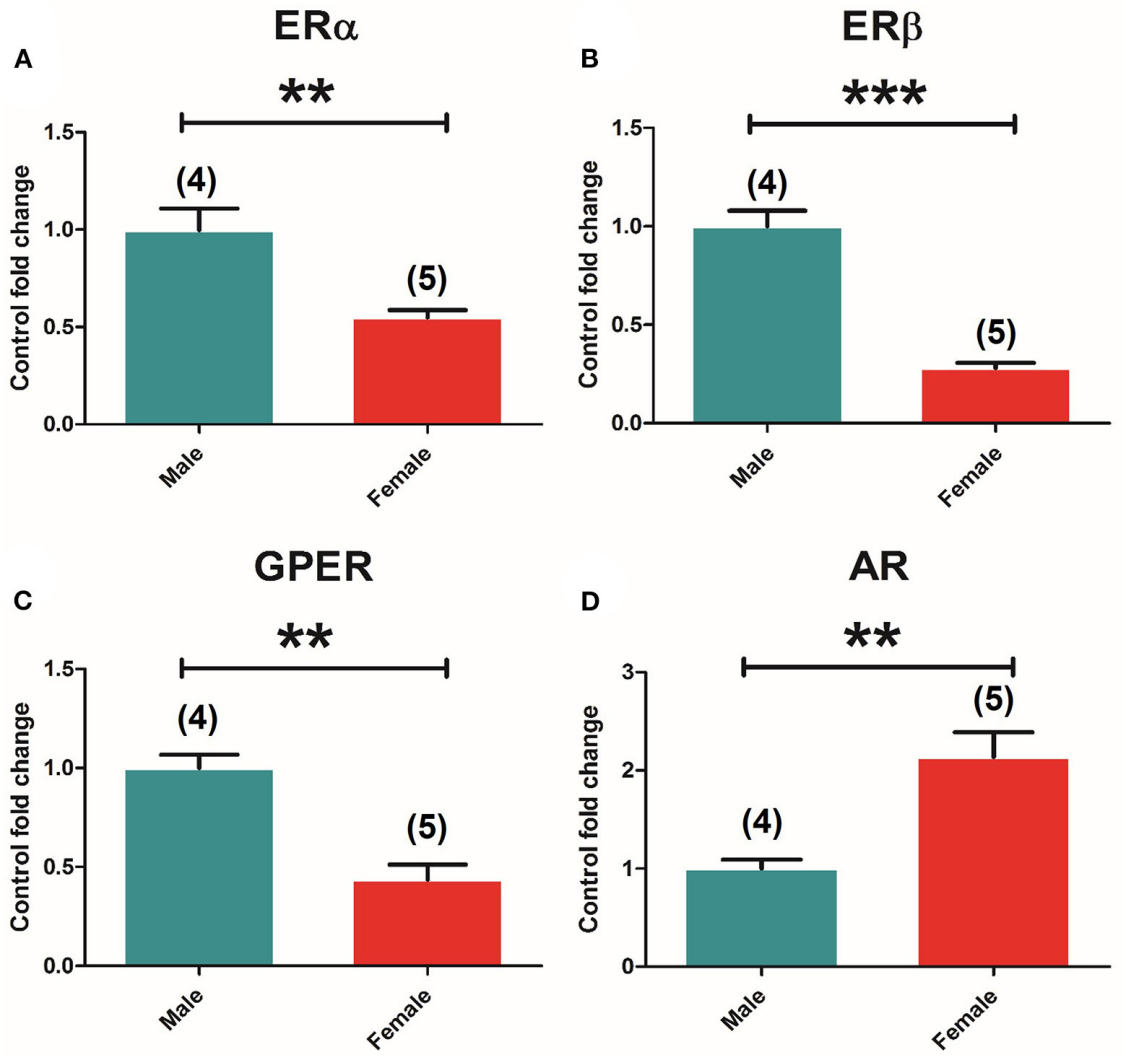
Sexual differences in ER and AR expression in the pituitary gland. **(A)** ERα mRNA levels. **(B)** ERβ mRNA levels. **(C)** GPER mRNA levels. **(D)** AR mRNA levels in male and female animals. Male animals (*n* = 4) and female animals (*n* = 5). Data are represented as mean ± SEM. **p < 0.01; ***Significant differences (***p < 0.001) vs. male control values.

Two-way ANOVA of the expression of the different receptors in the pituitary showed an effect of “sex” in all receptors, namely, ERα [*F*_(1, 37)_ = 14.263; *P* = 0.001], ERβ [*F*_(1, 37)_ = 113.691; *P* = 0.000], GPER [*F*_(1, 37)_ = 81.093; *P* = 0.000], and AR [*F*_(1, 37)_ = 28.415; *P* = 0.000]. The *t*-test comparison between control groups showed higher ERα [*t*_(7)_ = 4,289, *p* = 0.004] (Figure 3A), ERβ [*t*_(7)_ = 9,122, *p* = 0.000] (Figure 3B), and GPER [*t*_(7)_ = 5,541, *p* = 0.001] (Figure 3C) expression in males compared to females, and opposite results for AR [*t*_(7)_ = −3.873, *p* = 0.006], where females showed higher levels compared to males (Figure 3D).

### Flutamide, Finasteride, and Letrozole Exposure Modifies Estrogen and Androgen Receptor Expression in the Pituitary Gland

Selective inhibition of AR, 5α-reductase, and aromatase modulates ER and AR expression in the pituitary. Two-way ANOVA of receptors' expression showed a significant effect of early-time exposure to flutamide, finasteride, and letrozole in all receptors, namely, ERα [*F*_(3, 37)_ = 27.604; *P* = 0.000], ERβ [*F*_(3, 37)_ = 86.601; *P* = 0.000], GPER [*F*_(3, 37)_ = 91.684; *P* = 0.000], and AR [*F*_(3, 37)_ = 13.919; *P* = 0.000]. Subsequent Bonferroni's *post-hoc* comparison among controls and treated animals showed that flutamide decreased ERα (*P* = 0.007), ERβ (*P* = 0.000), and AR (*P* = 0.000), whereas flutamide increased GPER (*P* = 0.000) expression, finasteride decreased ERα (*P* = 0.000), ERβ (*P* = 0.000), and GPER (*P* = 0.001) and increased AR (*P* = 0.039) expression, and letrozole decreased all receptors' levels, namely, ERα (*P* = 0.000), ERβ (*P* = 0.000), GPER (*P* = 0.000), and AR (*P* = 0.000).

**Figure 4:**
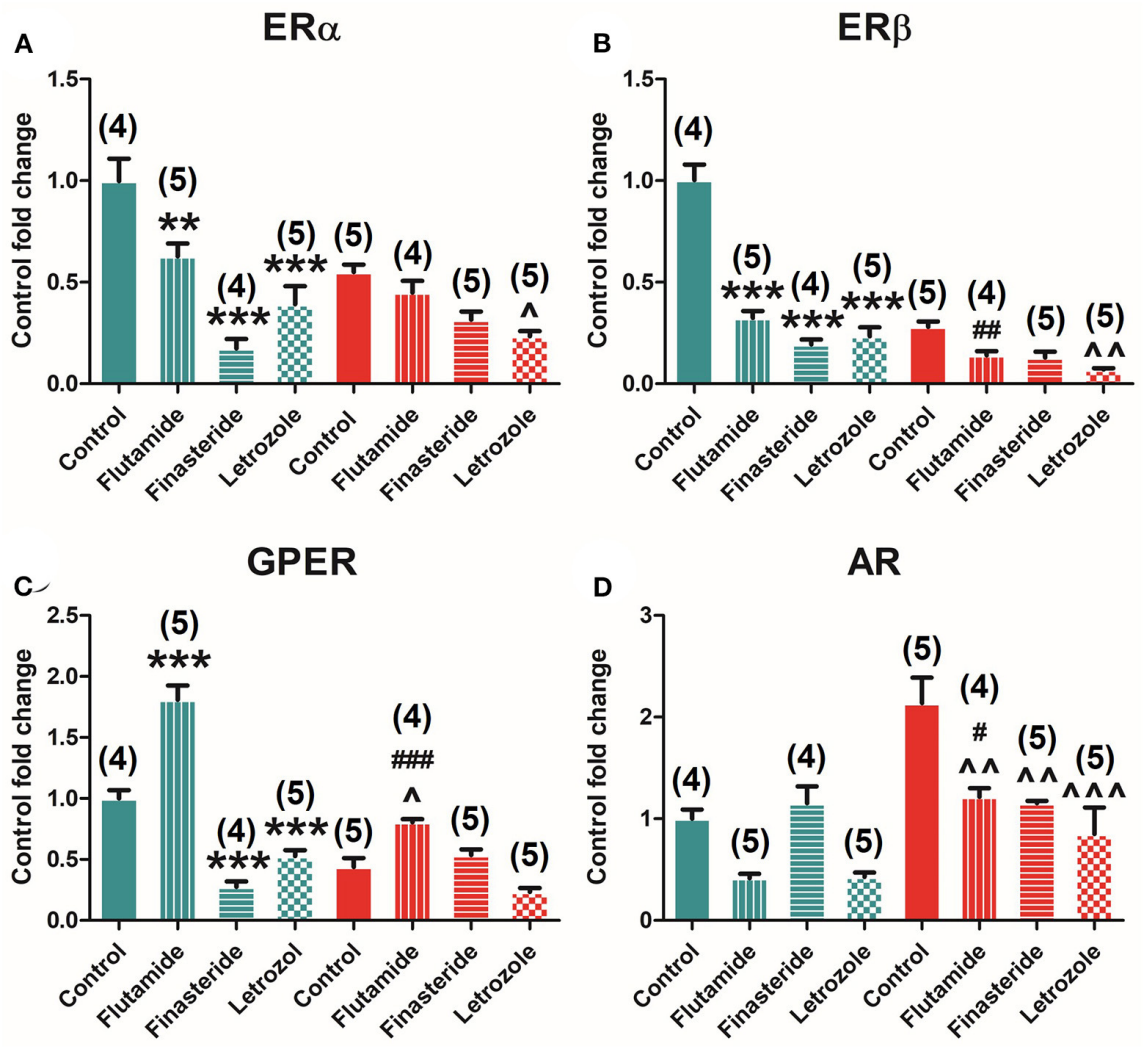
Effect of flutamide, finasteride, and letrozole exposure on ER estrogen and AR expression in the pituitary gland in male and female animals. **(A)** ERα mRNA. **(B)** ERβ mRNA. **(C)** GPER mRNA. **(D)** AR mRNA expression levels in male and female animals. Green bars represent male and red bars represent females. Control male (*n* = 4), flutamide male (*n* = 5), finasteride male (*n* = 4), letrozole male (*n* = 5), control female (*n* = 5), flutamide female (*n* = 4), finasteride female (*n* = 5), letrozole female (*n* = 5). Data are represented as mean ± SEM. **, ***Significant differences (**p < 0.01, ***p < 0.001) vs. male control values; ^∧^, ^∧∧^, ^∧∧∧^Significant differences (^∧^*p* < 0.05, ^∧∧^*p* < 0.01, ^∧∧∧^*p* < 0.001) vs. female control values; ^#^, ^##^, ^###^Significant differences (^#^*p* < 0.05, ^##^*p* < 0.01, ^###^*p* < 0.001) comparing female flutamide vs. male flutamide values.

Indeed, ERα was found to be negatively modulated by flutamide (*P* = 0.008), finasteride (*P* = 0.000), and letrozole (*P* = 0.000) treatments in male animals but only by letrozole (*P* = 0.025) in female animals (Figure 4A). Similar results found for ERβ levels in male animals were flutamide (*P* = 0.000), finasteride (*P* = 0.000), and letrozole (*P* = 0.000) treatments, decreasing ERβ expression, whereas slight differences were observed in female animals where finasteride (*P* = 0.014) and letrozole (*P* = 0.001) significantly reduced ERβ levels (Figure 4B). GPER expression was also detected to be negatively modulated by flutamide (*P* = 0.000), finasteride (*P* = 0.000), and letrozole (*P* = 0.001) treatment in male animals, whereas only flutamide exposure was able to modulate it, significantly decreasing its expression (*P* = 0.015) in females (Figure 4C). Finally, AR was found to be modulated only in female animals where flutamide (*P* = 0.004), finasteride (*P* = 0.004), and letrozole (*P* = 0.000) exposure induced a significant decrease in its expression (Figure 4D).

### Interaction Between Treatment and Sex Changes Dimorphism in Estrogen and Androgen Receptor Expression in the Pituitary Gland

A significant interaction between sex and treatment was found by two-way ANOVA in the expression of ERα [*F*_(3, 37)_ = 6.745; *P* = 0.001], ERβ [*F*_(3, 37)_ = 27.338; *P* = 0.000], GPER [*F*_(3, 37)_ = 32.017; *P* = 0.000], and AR [*F*_(3, 37)_ = 4.592; *P* = 0.009].

Moreover, a comparison among treated male and female animals showed that flutamide maintained dimorphism in the expression of ERβ (*P* = 0.036), GPER (*P* = 0.000), and AR (*P* = 0.02) but abolished dimorphism in ERα (Figures 4A–D). In contrast, both finasteride and letrozole abolished dimorphism in the expression of all ERs and AR (Figures 4A–D).

### Differential Estrogen and Androgen Receptors Expression in the Adrenal Gland

**Figure 5:**
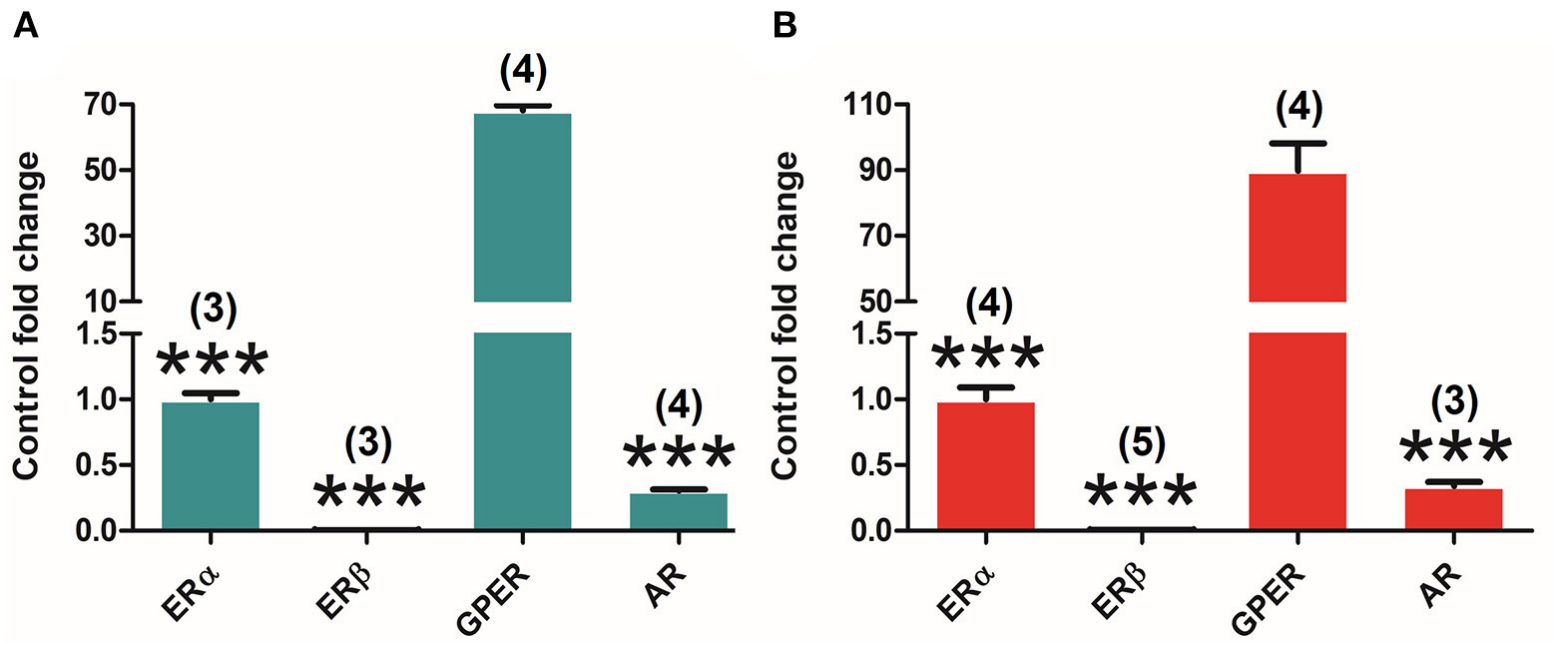
Differential ER and AR expression in the adrenal gland of adult male and female rats. **(A)** ERα (*n* = 3), ERβ (*n* = 3), GPER (*n* = 4), and AR (*n* = 4) expression levels in male animals. **(B)** ERα (*n* = 4), ERβ (*n* = 5), GPER (*n* = 4), and AR (*n* = 3) expression levels in female animals. Data are represented as mean ± SEM. ***Significant differences (***p < 0.001) vs. GPER values.

Both canonical (ERα and ERβ) and G protein-coupled ER (GPER) as well as AR are expressed in the adrenal gland of both adult male and female rats. Interestingly, the analysis of the expression levels of different receptors in the adrenal gland underlines differences in their relative amount in both males and females. One-way ANOVA of the receptors' relative amounts revealed significant differences in males [*F*_(3, 8)_ = 1941.544; *P* = 0.000], and subsequent Bonferroni's *post-hoc* test showed an extremely higher amount of GPER in comparison with ERα (*P* = 0.000), ERβ (*P* = 0.000), and AR (*P* = 0.000) (Figure 5A). Similar results were found in female animals [*F*_(3, 12)_ = 118.210; *P* = 0.000] where GPER displayed as well an extraordinarily higher expression compared to ERα (*P* = 0.000), ERβ (*P* = 0.000), and AR (*P* = 0.000) (Figure 5B). In both male and female animals, GPER seems to be the highest and ERβ the lowest expressed, showing ERα and AR intermediate levels of expression.

### Sex Dimorphism in the Estrogen and Androgen Receptors Expression in Adrenal Glands

**Figure 6:**
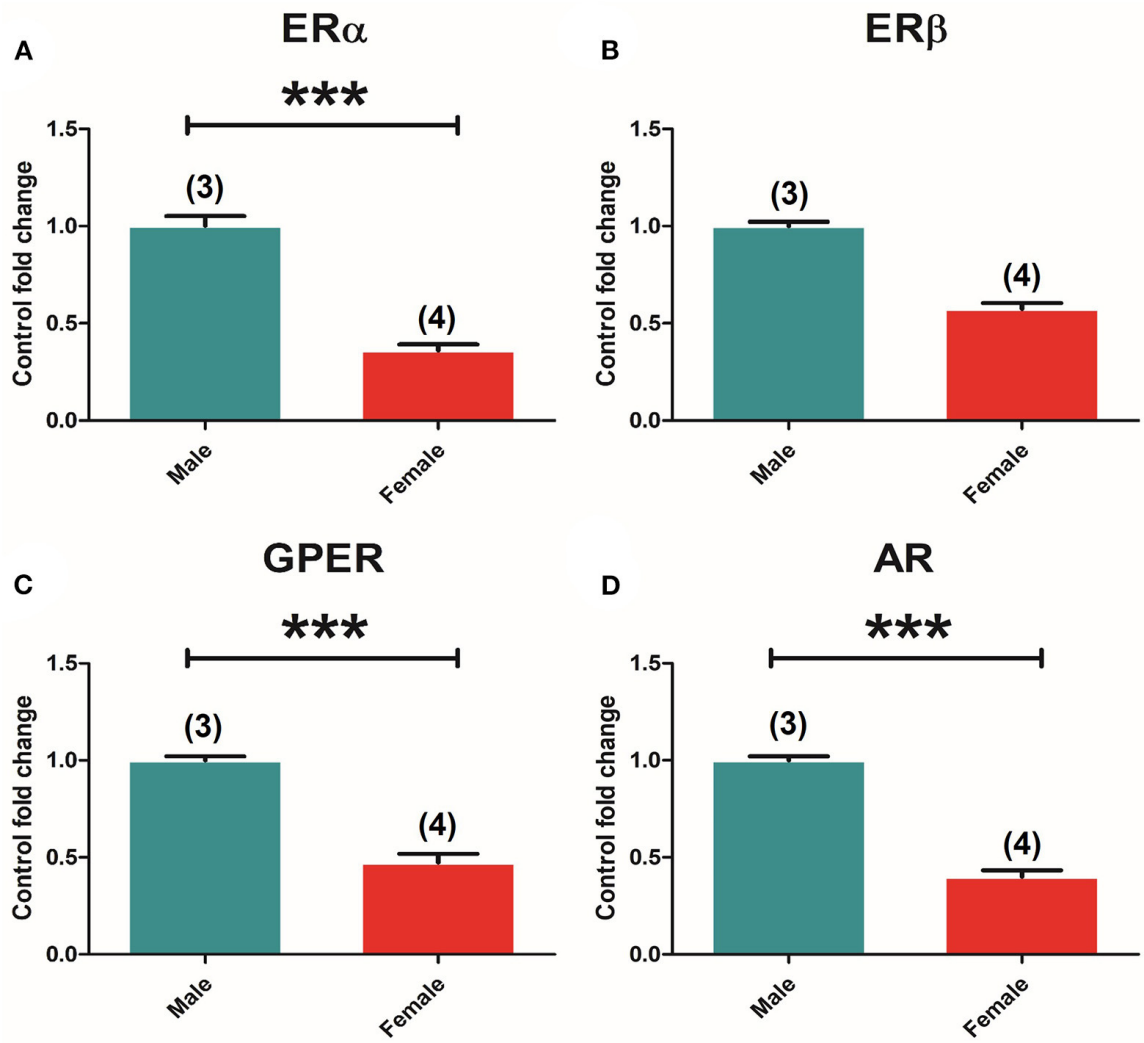
Sexual differences in ER and AR expression in the adrenal gland. **(A)** ERα mRNA levels. **(B)** ERβ mRNA levels. **(C)** GPER mRNA levels. Male animals (*n* = 3) and female animals (*n* = 4). **(D)** AR mRNA levels in male and female animals. Data are represented as mean ± SEM. *, ***Significant differences (*p < 0.05, ***p < 0.001) vs. male control values.

Two-way ANOVA of the receptors' expression showed a significant effect of “sex” in ERα [*F*_(1, 30)_ = 159.299; *P* = 0.000], GPER [*F*_(1, 30)_ = 121.837; *P* = 0.000], and AR [*F*_(1, 30)_ = 5.299; *P* = 0.031]. The *t*-test analysis of receptors' expression between male and female control animals revealed significant differences in ERα [*t*_(5)_ = 11,576, *p* = 0.000] (Figure 6A), GPER [*t*_(5)_ = 9,628, *p* = 0.000] (Figure 6C), and AR [*t*_(5)_ = 13,953, *p* = 0.000] (Figure 6D) levels in male compared to female animals, whereas ERβ seemed to show no differences between sexes (Figure 6B).

### Flutamide, Finasteride, and Letrozole Treatments Modify ERα, GPER, and AR Expression in Adrenal Glands

Selective inhibition of AR, 5α-reductase, and aromatase modulates ER and AR expression in the adrenal glands. Two-way ANOVA of receptors' expression after flutamide, finasteride, and letrozole treatments showed a significant effect in all receptors, namely, ERα [*F*_(3, 30)_ = 95.790; *P* = 0.000], ERβ [*F*_(3, 30)_ = 4.036; *P* = 0.020], GPER [*F*_(3, 30)_ = 11.000; *P* = 0.000], and AR [*F*_(1, 30)_ = 3.465; *P* = 0.034]. Subsequent Bonferroni's *post-hoc* test showed that flutamide and finasteride decreased ERα levels (*P* = 0.000), whereas letrozole decreased ERα (*P* = 0.000) and increased GPER expression (*P* = 0.000).

**Figure 7:**
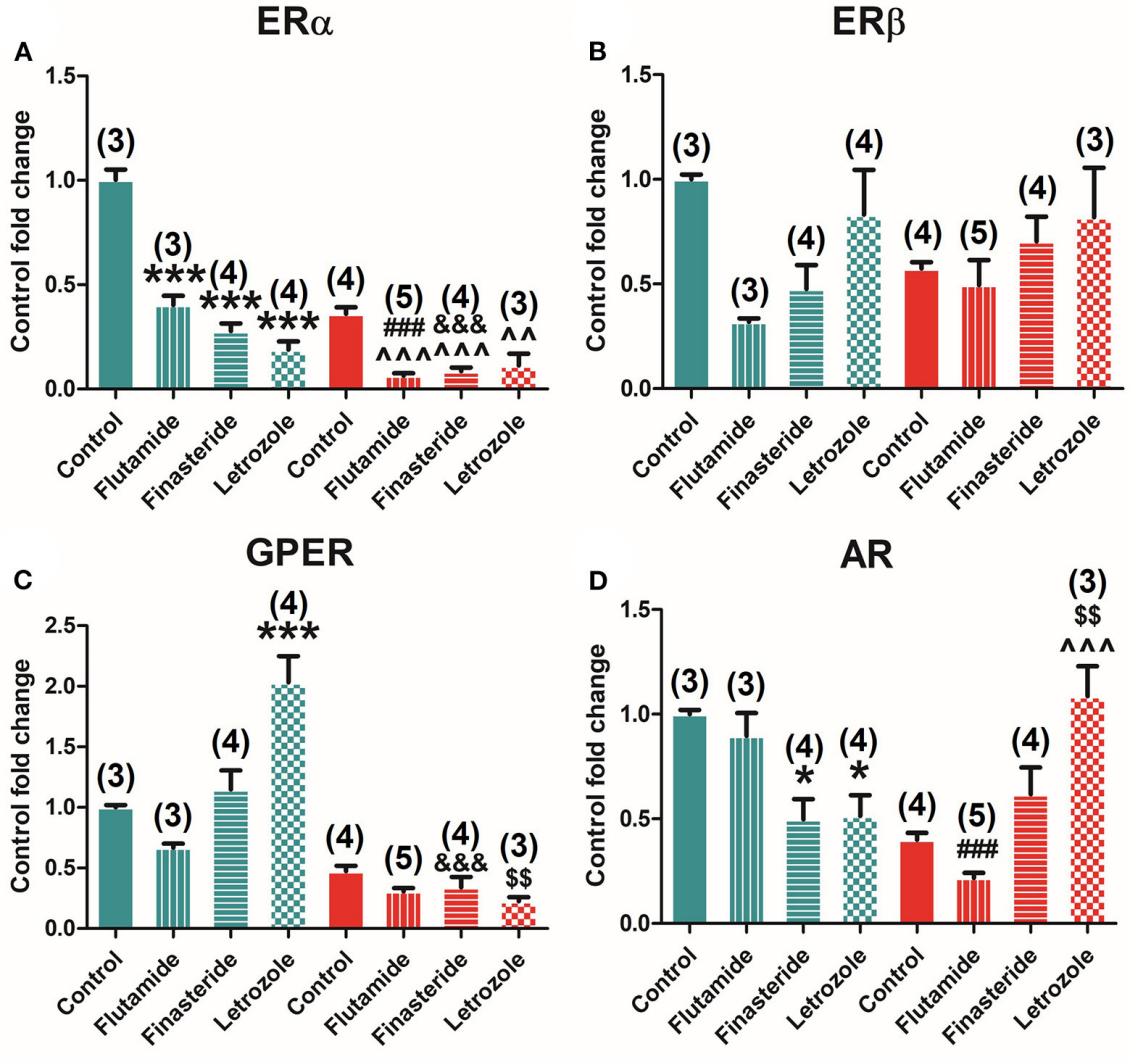
Effect of flutamide, finasteride, and letrozole exposure on ER and AR expression in the adrenal gland in male and female animals. **(A)** ERα mRNA. **(B)** ERβ mRNA. **(C)** GPER mRNA. **(D)** AR mRNA expression levels in male and female animals. Green bars represent males and red bars represent females. Control male (*n* = 3), flutamide male (*n* = 3), finasteride male (*n* = 4), letrozole male (*n* = 4), control female (*n* = 4), flutamide female (*n* = 5), finasteride female (*n* = 4), and letrozole female (*n* = 3). Data are represented as mean ± SEM. *, ***Significant differences (*p < 0.05, ***p < 0.001) vs. male control values; ^∧∧^, ^∧∧∧^Significant differences (^∧∧^*p* < 0.01, ^∧∧∧^*p* < 0.001) vs. female control values; ^###^Significant differences (^###^*p* < 0.001) comparing female flutamide vs. male flutamide values; ^&&&^Significant differences (^&&&^*p* < 0.001) comparing female finasteride vs. male finasteride values; ^$$^Significant differences (^$$^*p* < 0.01) comparing female letrozole vs. female letrozole values.

Indeed, ERα was found to be negatively modulated by flutamide (*P* = 0.000), finasteride (*P* = 0.000), and letrozole (*P* = 0.000) treatments in male animals and by flutamide (*P* = 0.000), finasteride (*P* = 0.000), and letrozole (*P* = 0.025) treatments in female animals (Figure 7A). GPER expression was detected to be modulated only by letrozole exposure (*P* = 0.000) in male animals, whereas no effects of treatment were displayed in female animals (Figure 7C). AR expression levels increased upon finasteride (*P* = 0.025) and letrozole exposure (*P* = 0.033) in male animals and just after letrozole exposure in female animals (*P* = 0.001) (Figure 7D). Finally, ERβ seemed to be unaffected by all treatments in both males and females (Figure 7B).

### Sex Dimorphism of AR in Adrenal Glands Is Modified by Finasteride and Letrozole

A significant interaction between sex and treatment was found by two-way ANOVA in the expression of ERα [*F*_(3, 30)_ = 23.174; *P* = 0.000], GPER [*F*_(3, 30)_ = 16.037; *P* = 0.000], and AR [*F*_(3, 30)_ = 21.889; *P* = 0.000].

In addition, comparisons among treated male and female animals showed that flutamide administration kept the dimorphism in ERα (*P* = 0.000) and AR (*P* = 0.000) expression, as well as finasteride for ERα (*P* = 0.000), but this latter additionally promotes dimorphism, not showed in controls, increasing GPER expression levels in male animals (*P* = 0.001) (Figures 7A–D). Finally, letrozole administration inverted the dimorphic expression of AR (*P* = 0.006) and abolished the sexual differences of ERα (Figures 7A–D).

## Discussion

Organizational effects of gonadal hormones during the first days of neonatal life have major irreversible effects on adults. Indeed, since very early in the postnatal development, they define sex-specific biological features that reflect sex-specific behaviors and physiology. In particular, testosterone exposure in the perinatal period has been shown to organize HPA axis functions in rodents, leading to the interesting idea that neonatal testosterone, either itself or *via* its metabolites E2 or DHT and interactions with estrogen or ARs (Goel and Bale, 2008; Goel et al., 2014), may modulate stress reactivity and sex-specific susceptibility to stress in adults. Our data demonstrate that selective blockage of either AR or aromatase or 5α-reductase from postnatal day 1 to postnatal day 5 alters the expression of ERs and AR in the pituitary and adrenal glands in adult male and female rats, strongly supporting the hypothesis of the major role of testosterone and its pathway and metabolites on the definition of the sex-specific biological features of the HPA axis.

We demonstrated here that ERα, ERβ, GPER, and AR are expressed in the pituitary gland of male and female adult rats, being ERα the highest and ERβ the lowest expressed, confirming and extending the previous data of Mitchner et al. (1998) and Okada et al. (2003), and that their expression is sexually dimorphic, being ERs levels higher in male animals and AR levels higher in female animals. Interestingly, testosterone, DHT, or E2 have been shown to be able to suppress GnRH-stimulated LH secretion from pituitary cultures (Frawley and Neill, 1984), whereas testosterone treatment seems to be able to increase basal FSH secretion and intrapituitary FSH levels (Kennedy and Chappel, 1985). Moreover, it has been demonstrated that estrogen responsiveness of the pituitary gland requires the presence of ERs, including ERα and ERβ (Demay et al., 1996).

The critical role of E2 in the anterior pituitary function has been reported particularly in PRL and gonadotropin gene transcription in both male and female animals. Indeed, ERα knockout mice showed decreased a PRL mRNA expression (Scully et al., 1997; Avtanski et al., 2014), an effect probably mediated by the interaction between ERα and the pituitary-specific transcription factor Pit-1 (Simmons et al., 1990). The somatotropic and lactotropic cells of the anterior pituitary originated from Pit-1 expressing progenitors (González-Parra et al., 1996; Scagliotti et al., 2021). Moreover, Pit-1 quantifications in the anterior pituitary at different postnatal days in male and female rats revealed sex differences within the first 10 days of age, with increased levels of Pit-1 mRNA in somatotrophs of males and increased levels of Pit-1 mRNA in lactotrophs of females (González-Parra et al., 1996). Moreover, six estrogen-responsive/ERα-dependent genes were identified in the gonadotropic cells of the pituitary, during proestrus in mice, suggesting that estrogen sensibilizes the pituitary to respond to gonadotropin-releasing hormone (GnRH) and increasing LH release (Kim et al., 2011). Supporting this, ERα knockout mice showed anovulation and infertility (Hewitt and Korach, 2003). Therefore, the highest ERα levels among the ERs, we found in both male and female animals, can support the fundamental role of ERα in the proper pituitary physiology and functionality of both sexes. Similar to ERα, GPER has been reported to be profusely expressed in anterior pituitary cells of female rats, particularly in lactotropic (Camilletti et al., 2019) and gonadotropic cells of male rodents where it seems to be involved in the modulation of the GnRH release as well as gonadotropins secretion (Chimento et al., 2014). Finally, it has been demonstrated that ERα is expressed in somatotropic cells of the anterior pituitary of female rats and it was described to regulate the transcriptional control of the GH (Avtanski et al., 2014). In contrast, the sexually dimorphic expression of ERα and GPER in the pituitary could be at the base of the sex differences in the stress response and stress-related diseases. Indeed, ERα is critical for PRL function that has been demonstrated to have a modulatory effect on reactivity to stress response and antidepressant treatment with increased PRL levels reported in a stress-resilience male rat phenotype (Faron-Gorecka et al., 2014) and decreased basal PRL plasma levels correlated with decreased responsiveness to antidepressant treatment (Faron-Gorecka et al., 2017). In addition, ovariectomy increased the GPER expression in lactotropic cells and GPER agonist treatment stimulated PRL secretion in female rats (Camilletti et al., 2019).

Finally, we have demonstrated a sexually dimorphic expression of AR, being this latter higher in female than male animals. Our data are supported by previous evidence showing an estrogen-dependent regulation of AR expression and cellular turnover. Indeed, cytosolic AR in the pituitary is higher during diestrus and proestrus in female rats (Handa et al., 1986) and E2 administration increased cytosolic ARs in the pituitary of ovariectomized females (Handa et al., 1987). Furthermore, castrated males increased cytosolic ARs in the pituitary in comparison with intact males, and DHT administration on castrated males disappeared differences with controls (Handa et al., 1986).

Gordon et al. described for the first time at the end of the 1970s that adrenal glands are the target of estrogens. Since then, other groups have reported the expression of ERα and ERβ (De Cremoux et al., 2008; Caroccia et al., 2016) GPER (Trejter et al., 2015) and AR (Antoniou-Tsigkos et al., 2000; Trejter et al., 2015; Gannon et al., 2020) in the adrenal glands. We have demonstrated here that GPER is the highest receptor expressed in the adrenal glands. Our results are in contrast to the study of Trejter et al. (2015) who described that ERα is the highest expressed. It is important to point out that they circumscribed their study to the adrenal cortex, not considering the adrenal medulla, which was demonstrated to be a relevant region of GPER localization (Hazell et al., 2009; Zheng et al., 2020). The specific role of GPER in adrenal glands is not yet fully understood but it seems that GPER-deficient rats showed a decreased corticosterone basal serum level in both sexes and an increased adrenaline basal serum level in females (Zheng et al., 2020). In addition, treatment with 17β-E2 increased catecholamines synthesis in cultured bovine adrenal medulla cells through a GPER-mediating mechanism, since inhibition of canonical ER was demonstrated to be ineffective on catecholamines synthesis (Yanagihara et al., 2006). Our data show that, in contrast to the pituitary, in the adrenal glands, GPER expression is not sexually dimorphic, which is in agreement with the study of Trejter et al. (2015).

Our data show that inhibition of aromatase or 5α-reductase during the first 5 neonatal days induced long-term effects of testosterone on the ERs and ARs expression in the pituitary. Indeed, a significant decrease in ERα and ERβ expression in male rats and of AR expression in female rats has been detected, abolishing the dimorphic expression of both ERs and AR in males and females, respectively. In contrast, inhibition of AR by flutamide exposure decreased ERα and ERβ in male to female values and decreased AR levels in female to male levels, abolishing in all the cases the dimorphic expression observed in control animals. These long-term effects of estrogens and DHT were able to feminize the ERα and ERβ expression in males and masculinize the AR expression in females. Interestingly, the ontogeny of the pituitary shows sex differences, i.e., GH mRNA levels have been found to be higher in males than females at birth (postnatal day 0), with a second period of divergent expression starting with the pubertal onset, and PRL mRNA levels are significantly higher in females starting with the pubertal onset (González-Parra et al., 1996). These developmental sex differences in the pituitary are gonadal steroid hormone dependent, since after birth, a peak of testosterone is present in males and during puberty, the concentration of gonadal steroid hormones changes in both sexes.

Our findings demonstrate that inhibition of aromatase or 5α-reductase during the first 5 neonatal days also affects the ERs and ARs in the adrenal glands. Indeed, a significant decrease in ERα in male and female animals, as well as of GPER in females and AR in males and an increase of GPER in males and AR in females, have been revealed. It has been demonstrated that, in the ontogeny of the adrenal gland, androgens start to be secreted by the adrenal cortex not earlier than the puberty, the same period that the gland starts to show a clear modification in the adrenal cortex of morphological and functional differences between male and female animals. Indeed, females show bigger cortex and zona fasciculata as well as increased plasma corticosterone levels. No significant differences between males and females have been reported at birth (Pignatelli et al., 2006). Interestingly, an early increase in adrenal androgen production can lead to premature adrenarche (Turcu et al., 2014). In addition, the evidence demonstrated that testosterone inhibits the HPA axis downregulating basal and stress glucocorticoid responses (Hodosy et al., 2012), whereas estrogen shows an opposite effect and that both effects are abolished by gonadectomy and restored by hormonal replacement treatment (Seale et al., 2004).

Our data demonstrate that aromatase blockage by letrozole in the perinatal days was able to invert the dimorphic expression of AR, bringing male AR levels to control female values and on the way around in males, suggesting a clear organizational function of the androgens in the ontogeny of the adrenal gland.

Perinatal administration of flutamide in males leads to a feminization of the HPA activity, increasing both corticosterone basal levels and stress response in male to female values (Seale et al., 2005b), and impairs stress response habituation maintaining elevated corticosterone response to repeated stress in comparison with controls and with gonadectomized animals supporting its organizational effect (Bingham et al., 2011). This is in agreement with our data showing that neonatal flutamide exposure decreased ERα levels in male to female values, fostering the idea that blockage of AR action during the development could impact the adrenal function in the adult males. In contrast, although finasteride and letrozole neonatal effects in the HPA axis are less explored, with less evidence that letrozole perinatal administration impairs hypothalamic masculinization in males (Gerardin et al., 2008) and finasteride neonatal administration produces an anxiogenic-like profile (Martin-Garcia et al., 2008); their impact in the masculinization of male HPA during critical developmental periods is supporting our results.

In general, we can conclude that inhibition of DHT and E2 production during the first-week critical period, thereby giving way to the organizing action of testosterone, mainly decreases the male ERs expression levels to female values and female AR expression to male levels with a net result of masculinization of the females and feminization of males in the pituitary gland and increases the AR expression in female to male levels, leading to masculinizing effects in the adrenal gland. In contrast, blockade of androgens' action, thus allowing the organizing action of estrogens, differentially modulates the ERs in males and females and decreases the AR in both males and females. Altogether, the data suggest that neonatal modifications of the androgen and estrogen pathways can potentially lead to permanent modifications of the neuroendocrine functions of the pituitary and adrenal glands in the adulthood of both sexes.

## Data Availability Statement

The raw data supporting the conclusions of this article will be made available by the authors, without undue reservation.

## Ethics Statement

The animal study was reviewed and approved by Institutional Bioethical Committee (UNED, Madrid).

## Author Contributions

DG, HP, and PC designed and supervised the experiments. NL, JF-G, NB, AB, BC, M-AA, HP, and DG performed the experiments. DG and NL prepared the figures for publication and wrote the first draft of the manuscript. All authors read and approved the manuscript.

## Funding

This study was supported by Agencia Estatal de Investigación, Spain, and Fondo Europeo de Desarrollo Regional (FEDER) (BFU2017-82754-R, PID2020-115829GB-I00, and PID2020-115019RB-I00), Universidad Autónoma de Madrid - Comunidad Autónoma de Madrid, Programa de estímulo a la investigación de jóvenes doctores (Project SI3-PJI-2021-00508), Centro de Investigación Biomédica en Red Fragilidad y Envejecimiento Saludable (CIBERFES), Instituto de Salud Carlos III, Madrid.

## Conflict of Interest

The authors declare that the research was conducted in the absence of any commercial or financial relationships that could be construed as a potential conflict of interest.

## Publisher's Note

All claims expressed in this article are solely those of the authors and do not necessarily represent those of their affiliated organizations, or those of the publisher, the editors and the reviewers. Any product that may be evaluated in this article, or claim that may be made by its manufacturer, is not guaranteed or endorsed by the publisher.
